# Post-processing sets of tilted CT volumes as a method for metal artifact reduction

**DOI:** 10.1186/1748-717X-9-114

**Published:** 2014-05-15

**Authors:** Hendrik Ballhausen, Michael Reiner, Ute Ganswindt, Claus Belka, Matthias Söhn

**Affiliations:** 1 Department of Radiotherapy and Radiation Oncology, Ludwig-Maximilians-University of Munich, Marchioninistrasse 15, 81377 Munich, Germany

## Abstract

**Background:**

Metal implants, surgical clips and other foreign bodies may cause ‘streaking’ or ‘star’ artifacts in computed tomography (CT) reconstructions, for example in the vicinity of dental restorations or hip implants. The deteriorated image quality complicates contouring and has an adverse effect on quantitative planning in external beam therapy.

**Methods:**

The potential to reduce artifacts by acquisition of tilted CT reconstructions from different angles of the same object was investigated. While each of those reconstructions still contained artifacts, they were not necessarily in the same place in each CT. By combining such CTs with complementary information, a reconstructed volume with less or even without artifacts was obtained. The most straightforward way to combine the co-registered volumes was to calculate the mean or median per voxel. The method was tested with a calibration phantom featuring a titanium insert, and with a human skull featuring multiple dental restorations made from gold and steel. The performance of the method was compared to established metal artifact reduction (MAR) algorithms. Dose reduction was tested.

**Results:**

In a visual comparison, streaking artifacts were strongly reduced and details in the vicinity of metal foreign bodies became much more visible. In case of the calibration phantom, average bias in Hounsfield units was reduced by 94% and per-voxel-errors and noise were reduced by 83%. In case of the human skull, bias was reduced by 95% and noise was reduced by 94%. The performance of the method was visually superior and quantitatively compareable to established MAR algorithms. Dose reduction was viable.

**Conclusions:**

A simple post-processing method for MAR was described which required one or more complementary scans but did not rely on any *a priori* information. The method was computationally inexpensive. Performance of the method was quantitatively comparable to established algorithms and visually superior in a direct comparison. Dose reduction was demonstrated, artifacts could be reduced without compromising total dose to the patient.

## Background

Planning in external beam radiotherapy critically relies on the ability to precisely delineate target volumes. As soft tissue contrast is limited, the image quality of the planning CT is often a limiting factor in the exact determination of boundaries and exacerbates inter-observer uncertainties. Also, the simulation of a plan demands a faithful reconstruction of the attenuation coefficient per voxel. The planning of intensity modulated radiotherapy and in particular proton and heavy ion therapy crucially depends on an unbiased representation of Hounsfield units.

In this context, metal implants such as dental restorations or hip prostheses give rise to beam hardening, scattered radiation, projection noise, trans-axial non-linear partial volume effects, and photon starvation, all of which may contribute to ‘streaking’ or ‘star’ or other artifacts that deteriorate the reconstruction in the vicinity of the foreign body [1]. A common workaround in clinical routine is to manually segment those artifacts slice by slice and to replace their voxel grey values by equivalent values for tissue, water, bone, air, respectively. If done by hand, this process is cumbersome, time consuming, and somewhat subjective.

Alternatively, artifacts can be suppressed during reconstruction. Metal artifact reduction (MAR) algorithms have been developed for this purpose [2-4]. These algorithms ideally operate on the raw sinogram data, but may use forward-projected ‘virtual sinograms’ instead [5]. In general, some assumption on the missing information is necessary, and typically the metal-affected parts of the sinogram are replaced by interpolation. For example, metal is segmented in the image space of each uncorrected slice and the ‘metal map’ is forward-projected to sinogram space. Then, the affected areas are replaced by linear one-dimensional interpolation ( [2], referred to as LI-MAR in this paper). The interpolation may be bi-directional within a slice and weighted ( [6,7], reffered to as BI-MAR in this paper) or even comprise interpolation across slices over the longitudinal scan range [8]. The method can be extended by normalizing the sinograms ( [9,10], referred to as NMAR) and by combining the high frequencies of the original reconstruction (for sharper edge rendering) with the low frequencies of a MAR-processed image ( [11], referred to as FSMAR). Alternatively, the in-painting may be based on a tissue-class model and an automatic segmentation of the initial reconstruction [12]. This resembles the discrete in-painting by hand as described above, but the blending takes place in sinogram space. Adaptive filtering techniques have also been proposed [13], as well as the replacement of the filtered back-projection by algebraic solvers [14-17]. Hybrid approaches combining or iterating the above elements have also been suggested [18,19]. Still, metal artifact reduction remains an open problem in clinical routine.

## Methods

**Figure 1:**
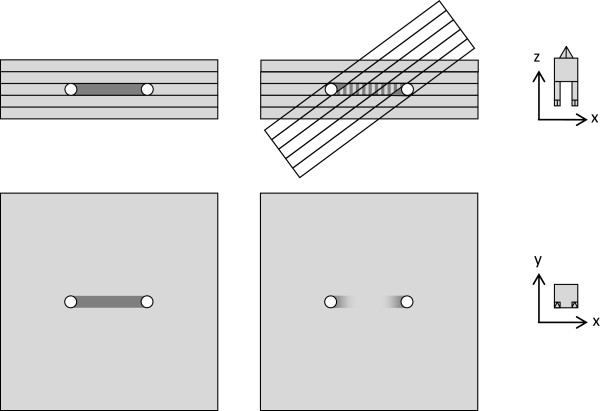
**An additional tilted CT provides complementary information.** Left top (coronal plane): a reconstructed CT consists of a stack of slices (light grey bars), and one of the slices contains two opaque bodies (white circles). Left bottom (transverse plane): this slice containing the opaque bodies is prone to feature a streaking artifact between them (dark grey area). Right top (coronal plane): An additional CT which is tilted against the first one offers complementary information from a different angle, in particular, the dark grey area is partially covered by slices which do not contain any opaque bodies. Right bottom (transverse plane): as a result, when averaging over two or more CTs with complementary information, streaking artifacts are reduced.

The aim of this study was to investigate an alternative approach. The question was if metal artifacts could be suppressed or removed in a post-processing step by comparing and combining two or more CTs of the same object which had been acquired at different angles. When comparing such CTs, one would observe that the location and orientation of the artifacts was not static. Rather, such redundant CTs would provide complementary information, where some regions were free of artifacts in one CT, and other regions were free in another.The basic idea was then to acquire a series of such CTs, ‘tilted’ against one another, and realign the set of reconstructed volumes. Then, for each voxel, the grey value would be computed from e.g. the median or average of the respective voxels in all co-registered CTs. Ideally, this estimate would be free of or less prone to artifacts than each individual CT on its own, see Figure 1.

As a beneficial side effect, one would expect overall image quality to improve. First, noise would be reduced when summing over several exposures. And second, aliasing from finite slice thickness would be reduced because tilted CTs could be resampled to a mesh of finer resolution.

The general concept to reduce noise by averaging over independent data is one of the paradigms of quantitative science, and also in imaging related fields the idea to reduce both noise and artifacts by super-sampling is well established. Examples comprise but are not limited to resonance Raman spectroscopy [20,21], optical coherence tomography [22] and magnetic resonance imaging [23,24]. In the narrower context of metal artifact reduction algorithms, hybrid algorithms such as [17] and [18] can be viewed as averaging over (semi-independent) data. Still, to our knowledge, the notion to reduce metal artifacts in computerized tomography by averaging over reconstructions independently acquired from different angles is new.

### Facility-specific technical information

All CTs were acquired by a standard Toshiba Aquilion LB scanner, used routinely as the planning CT in our clinic. Standard settings for head and neck tomography were used. Resolution was (0.1 cm)^3^ per voxel. Volumes were reconstructed by the filtered back-projection algorithm of the integrated software suite of the CT scanner. Reconstructed volumes were stored as 16 bit Digital Imaging and Communications in Medicine (DICOM) on the server for post-processing.

**Figure 2:**
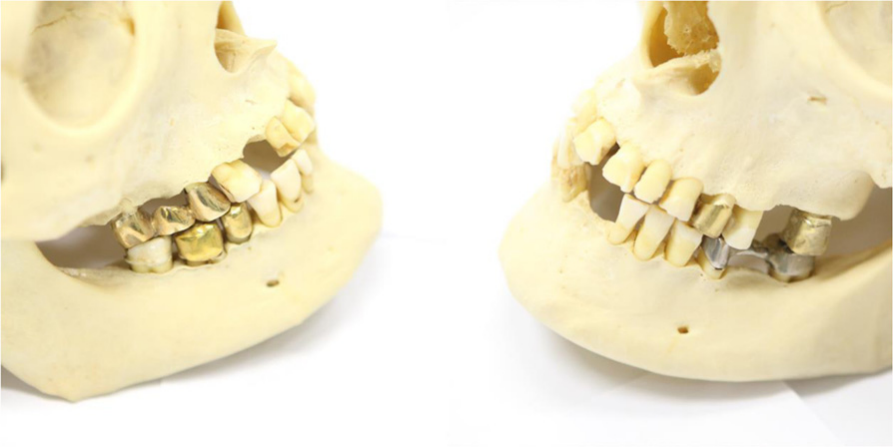
**Dental implants are a typical cause of head and neck metal artifacts.** A human skull was used as a ‘worst case’ test object for metal artifact reduction. It features no less than six separate metal foreign bodies: a full gold bridge, a full steel bridge, and four full gold crowns.

The first test object was a tissue characterization phantom. The Gammex 467 phantom consisted of a disc of 33 cm diameter and 5 cm height made from ‘Solid Water’ water equivalent material. A recent characterisation of the material [25] under identical circumstances (same Gammex 467 phantom, same Toshiba Aquilon LB) in comparison to water and other ‘water equivalent’ materials found a CT density of 11 Hounsfield units. The phantom featured 16 holes of 3 cm diameter which were filled with cylindrical insets consisting of materials of varying Hounsfield density. In particular, the configuration used in the experiment included a titanium insert featuring an electron density 3.79 times that of water [26], corresponding to 2790 Hounsfield units. Titanium attenuates 80 keV photons by 0.405 cm^2^ g^-1^[27]. At a physical density of 4.59 g cm^-3^[3] this corresponds to an attenuation coefficient of 1.86 cm^-1^ permitting less than 1% of photons to pass 3 cm of travel distance. One may expect severe artifacts due to ‘photon starving’ around the titanium insert, in particular streaking artifacts in direction of other insets with high electron density.The second test object was a human skull featuring several missing teeth and dental restorations in the upper and lower jaw. Specifically; teeth 13, 16, 18, 26, 28, 31, 38, 41, 46 and 48 were missing; there was a full gold bridge from tooth 15 to tooth 17, there were full gold crowns on teeth 24, 27, 35 and 36; there was a full metal bridge from tooth 45 to tooth 47, see Figure 2. Due to this multitude of implants, the jaw presents an especially challenging test case for metal artifact reduction.

### Experimental procedure

**Figure 3:**
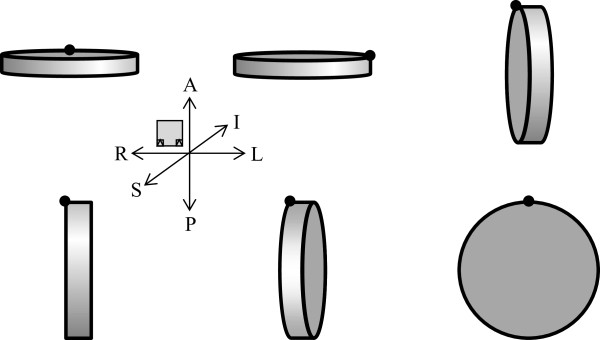
**The Gammex phantom was scanned in a total of six positions.** The Gammex phantom was first scanned lying flat on the patient table being turned by 0° and 90° about the vertical axis (top left and top middle), then standing standing upright being turned by 135°, 90°, 45° and 0° about the vertical axis (top right and bottom row).

The Gammex phantom was scanned in a total of six positions, see Figure 3. First lying flat being turned by 0° and 90° about the vertical axis, then standing upright being turned by 135°, 90°, 45° and 0° about the vertical axis. The skull was scanned in a total of nine positions, being turned by 0°, ±22.5°, ±45°, ±67.5° and ±90° about the vertical axis.

In a second experiment about possible dose reduction, a single scan of the Gammex phantom was acquired at 400 mA/s, or 200 mA per cycle of 0.5 seconds, respectively. This scan was repeated eight times at 1/8th of dose, at 50 mA/s or 25 mA per cycle. For comparison, further eight scans at 50 mA/s or 25 mA per cycle were acquired while the phantom was rotated by 0°, ±22.5°, ±45°,±67.5°, and +90° about the vertical axis between scans in order to provide a set of independent scans for the MAR method.

Noise levels and severity of artifacts were compared between the single scan at full dose, the sum of the former eight scans at reduced dose, and the median of the latter eight scans after co-registration.

### Data processing

For co-registration, the reconstructed input volumes were rotated back and shifted back into a common output mesh of resolution (0.1 cm)^3^ in the laboratory frame of reference. In order to do so, the coordinates of any voxel in any input volume were transformed via a rotation matrix and a translation vector. The resulting non-integer coordinates were mapped to integer coordinates in the output volume, and the residual coordinate fractions were used for tri-linear sampling. Rotation angles were measured during the experimental setup and minimally corrected ex post for optimal match of the volumes. Sub-voxel alignment was performed through the translation vector to +/-0.5 voxels in each direction by visual cues alone. Non-rigid registration was also tested but did not provide better alignment due to the solid nature of the samples. For patients, non-rigid co-registration may be needed, probably after a preliminary co-registration as described above. After co-registration, the per-voxel-mean and per-voxel-median of the matching volumes was calculated and exported for evaluation. Co-registration and mean/median calculations were performed by an in-house developed algorithm implemented in C#. The implementation resembled the following pseudo-code (for the average, the median is trivially similar):

### Evaluation

Three different measures for the impact of an artifact on the grey values of a given region of voxels are considered: “Bias”, “Error” and “Noise”. All three are conveniently measured in Hounsfield units. “Bias” is the overall increase or decrease of average grey value. “Error” is the average total deviation of each voxel from its true value. “Noise” is the standard deviation of voxel grey values, see [5]. “Bias” and “error” require a knowledge of true grey values, “noise” is meaningful in case of a region of constant grey values. The three measures are quantitatively defined as follows:

Consider some sub-volume consisting of *n* voxels, *i* = 1, …, *n*. Assume that the true Hounsfield units in this sub-volume are known and constant *c*. Denote the grey value of voxel *i* of the reconstructed volume *x*_*i*_. “Bias” is defined as *E*_*Bias*_ = *x*_*i*_ - *c*. “Error” is defined as *E*_*Error*_ = |*x*_*i*_ - *c*|. “Noise” is defined as the square root of $E_{\mathrm{Noise}}^{2}=\frac{1}{n-1}\sum {\left(x_{i}-x_{i}\right)}^{2}$ . In case of the Gammex phantom, a ring-shaped area around the titanium insert is considered with inner diameter 8 mm and outer diameter 16 mm. A stack of 7 mm of the innermost slices contains *n* = 4172 voxels in this area. This region is filled with ‘Solid Water’ and *c* = 11 *HU* is assumed, according to [25]. Results for a generic assumption of *c* = 0 *HU* would be quantitatively similar.

In case of the human skull, a volume of 6.1 × 1.1 × 4.1 cm^3^ centred in the mouth cavity between the dental implants is considered. This region is entirely filled with air which has *c* = - 1000 *HU* by definition. Note that this is represented digitally by zero values, so *E*_*Bias*_ and *E*_*Error*_ coincide. Results were checked to be similar for other volumes outside the skull close to the dental implants.

### Comparison to other algorithms

The performance of the method was compared to four established algorithms: linear interpolation of the metal-affected areas of the sinogram ( [2], referred to as LI-MAR in this paper), a bi-directional interpolation of the sinogram looking for close-by unaffected pixels (both in terms of axis and angle) and weighting them by range ( [6,7], reffered to as BI-MAR in this paper), normalized MAR ( [9,10], referred to as NMAR), and frequency split MAR ( [11], referred to as FSMAR). In all cases a virtual sinogram [5] was generated from the original reconstruction and used as input to the algorithms. Straightforward, non-optimized implementations were programmed. The threshold for the computation of metal maps was 2000 HU. In case of FS-MAR, the low frequency image was generated by Gaussian blurring with a radius of 3 pixels, and a blurred version of the metal map with Gaussian radius of 30 pixels was used as blending weight. A computation in debug mode on consumer hardware (laptop, single processor, single-threaded execution on a single core running at 2.6 GHz) allowed for a rough comparison of computing times. The quantitative performance of all algorithms was measured in terms of bias, error and noise as above. The quality of the reconstruction was compared visually, too.

## Results

### Phantom (‘Gammex’)

**Figure 4:**
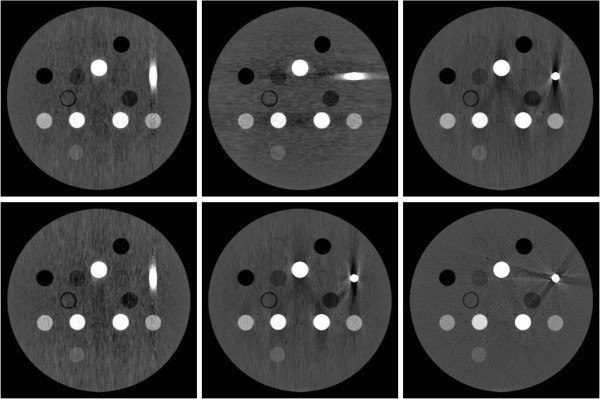
**The location of artifacts depends on the orientation of the phantom: ‘tilted’ CTs from different angles provide complementary information.** Six reconstructions of the same Gammex 467 phantom have been obtained from CTs taken at different angles. After co-registration, the reconstructions may be compared side by side as in this figure. In each case, the titanium insert on the right side causes streaking artifacts. However, the regions affected (or unaffected) by artifacts depend on the gantry angle. Therefore, the different reconstructions provide complementary information.

Figure 4 shows the six unprocessed reconstructions of the Gammex 467 phantom after co-registration. In each case, the titanium insert causes streaking artifacts. However, the regions affected (or unaffected) by artifacts depend on the orientation of the phantom relative to the gantry. Obviously, the different reconstructions provide complementary information.

**Figure 5:**
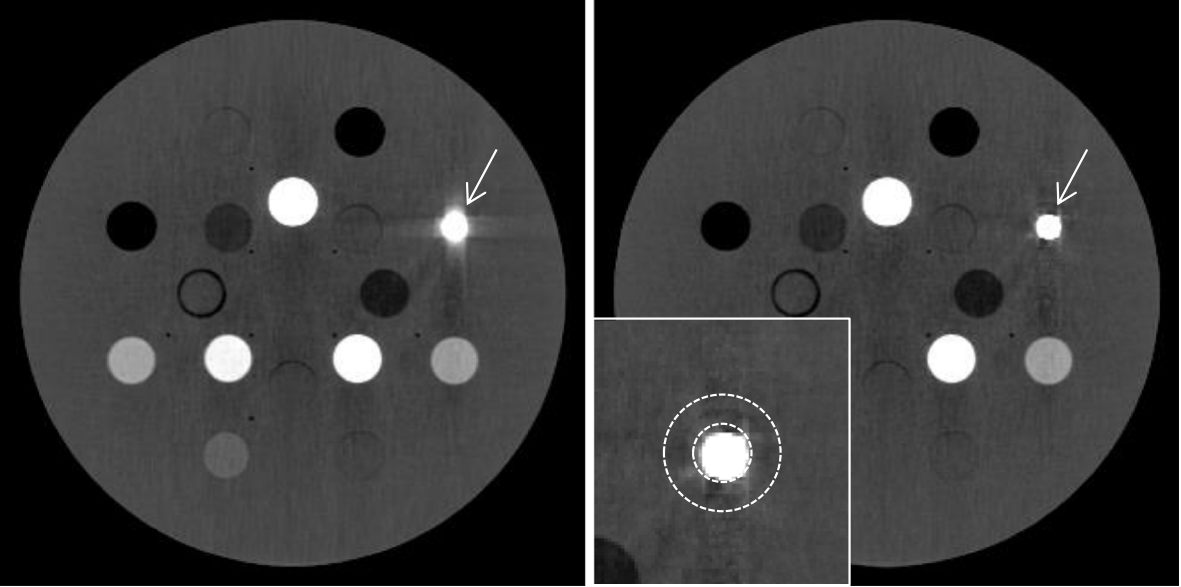
**Metal artifacts are clearly suppressed.** Left: Mean of the six reconstructions in Figure 3. The streaking artifacts are still visible, but to a lesser degree. Right: Median of the six reconstructions in Figure 3. The streaking artifacts are strongly suppressed. In both cases, details in the vicinity of the metal inset become much more visible, e.g. the thin gap of air around the inset (arrow); in comparison to a single reconstruction, noise is reduced as an additional benefit. The inset shows the ring-shaped region of interest for the calculation of bias, error and noise around the metal body.

For example, the two constructions on the left feature a ‘positive’ artifact in a vertical direction, while the middle bottom and right top reconstructions feature a ‘negative’ artifact in the same direction. One may hope that these artifacts will cancel to some degree in the average of the reconstructions. Also, no sub-region is affected by aretefacts in more than four reconstructions out of the six simultaneously. Hence artifacts will be suppressed in the median of the reconstructions.Figure 5 shows the mean (left) and the median (right) of all six reconstructions. In case of the mean, the streaking artifacts are still visible, but to a lesser degree. In case of the median, the streaking artifacts are strongly suppressed. In both cases, details in the vicinity of the metal inset become much more visible, e.g. the thin gap of air around the inset (indicated by the arrow in the figure). Also, in comparison to a single reconstruction, noise is reduced as an additional benefit.

**Figure 6:**
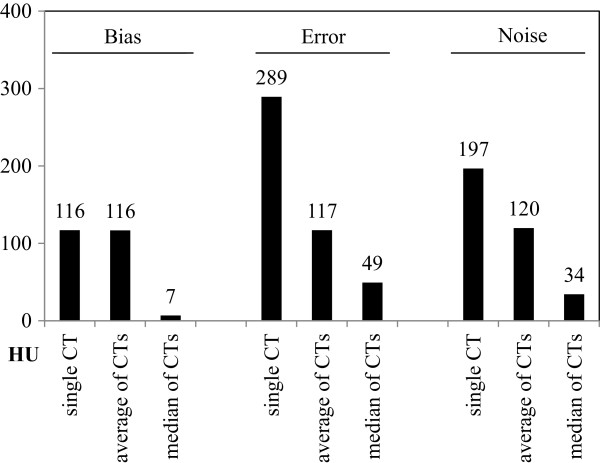
**Bias is reduced by 94% and noise is reduced by 83%.** Bias (left) is reduced from 116 HU on average to 6.6 HU in case of the median of six CTs. Similarly, per-voxel-error (middle) and noise (right) are reduced from 289 HU resp. 197 HU in case of a single CT (on average) to 117 HU resp. 120 HU in case of the mean of six CTs and even to 49 HU resp. 34 HU in case of the median of six CTs.

Qualitatively, the processed image shows significantly less artifacts, and even the thin air filled gap immediately around the titanium insert becomes visible (indicated by the arrow). Noise is reduced throughout the image because of super-sampling.Quantitatively, in the six individual CTs, the bias in the region around the titanium insert ranges from -36 HU to +262 HU. The average bias of 116 HU is reduced by 94% to 6.6 HU in case of the median, see Figure 6 (left).The average voxel error ranges from 66 HU to 299 HU in the six individual CTs, 197 HU on average. The average of the six CTs improves this figure by 39% to 120 HU, and the median reduces average pixel error by 83% to 34 HU, see Figure 6 (middle).Noise ranges from 83 HU to 458 HU in the six individual CTs, 289 HU on average. The mean reduces this noise by 60% to 117 HU, and the median reduces the noise by 83% to 49 HU, see Figure 6 (right).

### Human skull

**Figure 7:**
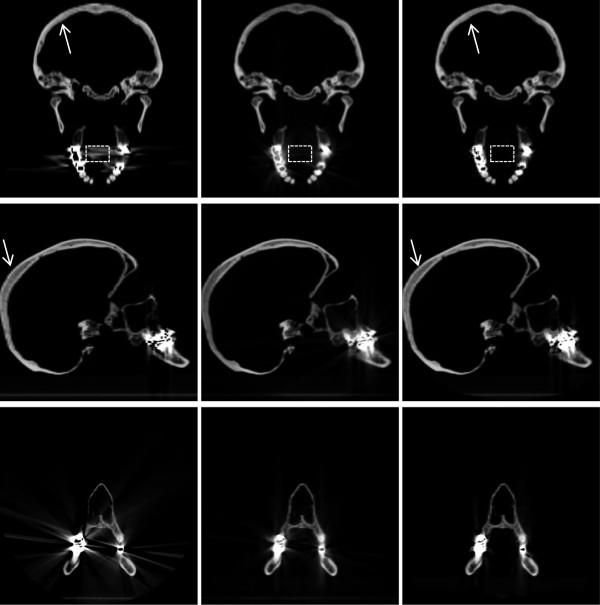
**Image quality and visible detail are clearly improved.** Comparison of a single CT (left) to the average (middle) and median (right) of nine tilted CTs: streaking artifacts are strongly reduced, especially in the transverse and coronal view. Details in the vicinity of the metal implants are more truthfully represented, e.g. grey values of bone tissue within the bridges and crowns. In addition, aliasing has been reduced as well, which is especially visible in the occipital bone (arrows), a beneficial side effect of averaging over multiple exposures from different angles. The bounding box shows the region of interest for the calculation of bias, error and noise centered in the oral cavity.

Figure 7 shows cross-sections of the unprocessed volume reconstruction of the human skull (left) in comparison to the corresponding cross-sections through the mean (middle) and median (right) voxel data of nine independent reconstructions. Visually, the reduction of streaking artifacts is obvious. At the same time, details in the vicinity of the metal restorations become much more visible.

**Figure 8:**
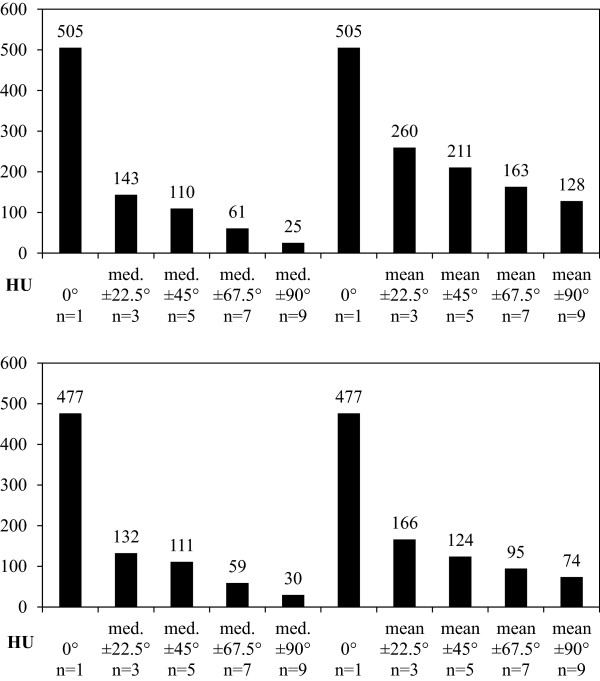
**Bias and noise are reduced by up to 95%.** Top: Bias is defined as the difference between average voxel value and -1000 HU and is calculated for the median (left) and mean (right) of a number of CTs. As more CTs with complementary information from an increasing range of angles are added, bias is reduced by 95% in case of the median resp. 75% in case of the mean. Bottom: Noise is defined as the standard deviation of voxel grey values in HU and is calculated for the median (left) and mean (right) of a number of CTs. As more CTs with complementary information from an increasing range of angles are added, noise is reduced by 94% in case of the median resp. 85% in case of the mean.

As an additional benefit, the super-sampling involved suppresses the aliasing due to finite slice thickness visible in the original image, which is particularly visible at the occipital bone.Quantitatively, the bias in the mouth cavity, which is air filled and should ideally feature -1000 HU, is reduced from 505 HU by 95% to 25 HU in case of the median of the nine CTs, and by 7% to 128 HU in case of the mean of the nine CTs. Noise is similarly reduced by 94% in case of the median, and by 85% in case of the mean, see Figure 8.Moreover, Figure 7 shows the incremental reduction of bias and noise as more CTs with complementary information from an increasing range of angles are added. The reduction of bias and noise from only three CTs taken at -22.5°, 0° and +22.5° is already substantial at 72%.

### Dose reduction

In the above experiment with the Gammex phantom, 197 HU noise were encountered at 50 mA/s or 25 mA per cycle. If current were eight times higher at 400 mA/s or 200 mA per cycle, noise levels were expected to decline to 197 HU / √8 ≈ 69 to 70 HU. In fact, in a second experiment with the same phantom, same experimental setting, 70 HU noise were recorded in a single scan at 400 mA/s or 200 mA per cycle (same region of interest in the reconstructed volume as before). Similarly, the scan was repeated eight times at 50 mA/s or 25 mA per cycle, and a volume was added up from the eight reconstructed volumes. In this average volume, noise of 78 HU was detected, of the same order of magnitude.

Finally, the eight scans were repeated at 50 mA/s or 25 mA per cycle, but this time the phantom was rotated between scans to acquire eight reconstructions from different angles as needed for the MAR method. In case of the co-registration based on the mean, noise level was 45 HU and in case of the co-registration based on the median, noise level was 60 HU. Noise levels are lower because of the non-cubic voxel dimensions which lead to effective anti-aliasing when rotated back and co-registered. At the same time, artifacts were reduced by 77.5% in terms of bias when compared to the single scan at 400 mA/s or 200 mA per cycle. Note that artifacts were not reduced at all when comparing the single 400 mA/s scan with the additive eight 50 mA/s scans, as expected. In conclusion, the MAR method did accomplish a significant reduction of artifacts, while retaining or even slightly improving noise levels, at constant overall dose.

### Comparison to established algorithms

**Figure 9:**
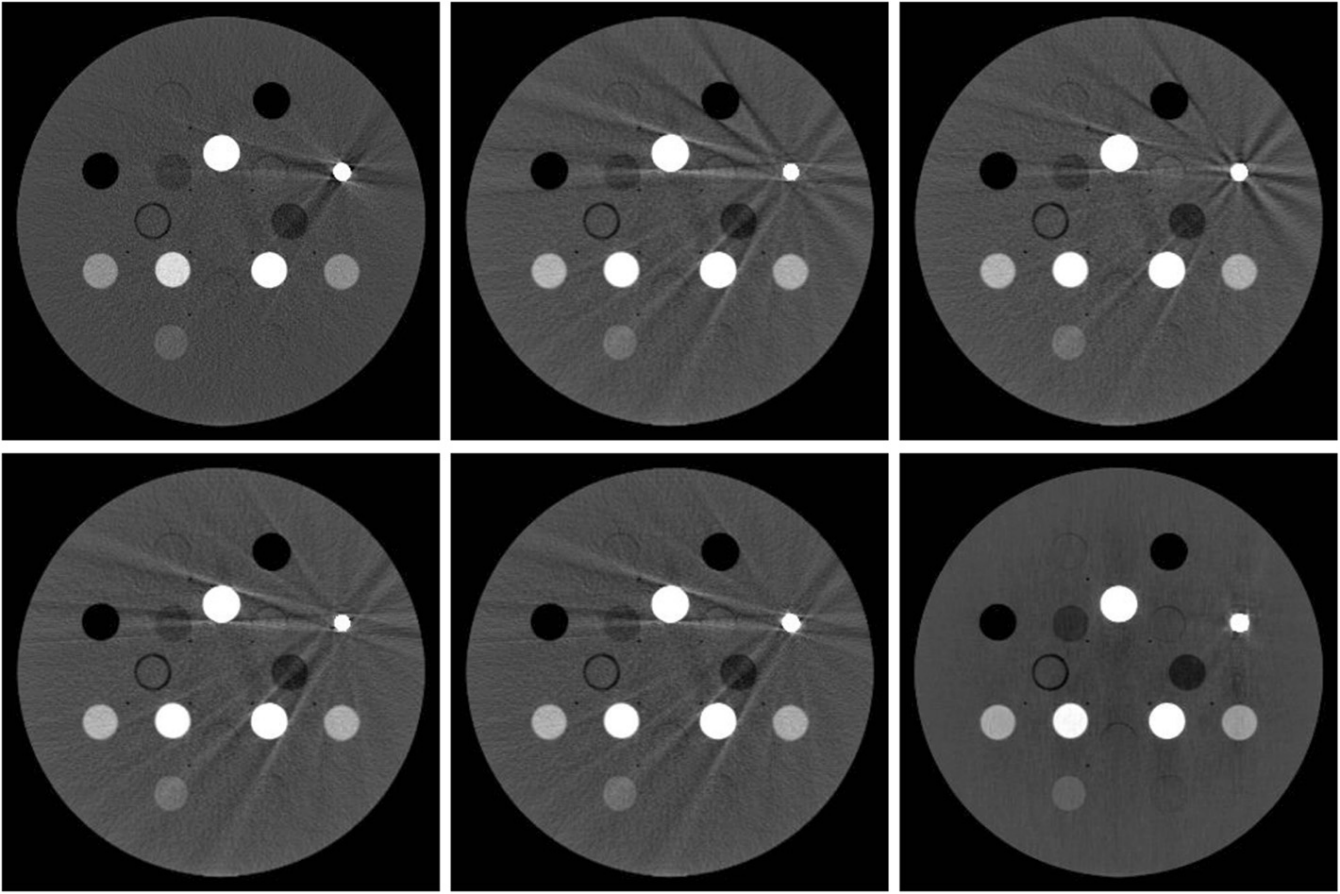
**Comparison to other MAR algorithms.** Top left: uncorrected filtered back-projection (FBP), top middle: linear interpolation MAR (LI-MAR, [2]), top right: bilinear interpolation MAR (BI-MAR, [6,7]), bottom left: normalized MAR (NMAR, [9,10]), bottom middle: frequency split MAR (FSMAR, [11]), bottom right: this paper.

**Figure 10:**
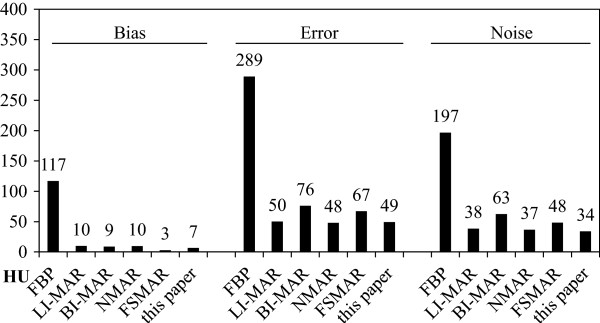
**Performance compared to other MAR algorithms.** The performace of the proposed method was compared to the uncorrected filtered back-projection (FBP), to linear interpolation MAR (LI-MAR, [2]), to bilinear interpolation MAR (BI-MAR, [6,7]), to normalized MAR (NMAR, [9,10]), and to frequency split MAR (FSMAR, [11]). Overall, the method performed as well as the established algorithms. FSMAR featured less bias, still in terms of error and noise the proposed method yielded one of the better results.

In a direct visual comparison to established MAR methods, the described method featured less visible artifacts and noise, see Figure 9. LI-MAR, BI-MAR, NMAR and FSMAR all introduced some new artifacts at higher frequencies but of less amplitude than the original. This was probably due to the use of a virtual sinogram as the newly introduced artifacts were aligned with the edges of other opaque bodies, a result of the additional forward-projection and filtered back-projection. It was assumed that the methods would have performed better on original sinogram data, which was unavailable. In case of the described MAR method, which does not rely on virtual sinograms, no new artifacts were introduced. Noise was substantially lower in case of the described method, but this could be attributed to oversampling and less so to very minor blurring due to tri-linear sampling.Quantitatively, artifacts were reduced by all methods, see Figure 10. Bias was reduced from 117 HU to 9 to 10 HU in case of LI-MAR, BI-MAR and NMAR. FSMAR performed even better at only 3 HU. The described method was second best at 7 HU. Error was reduced from 289 HU to about 48 to 50 HU in case of LI-MAR, NMAR and the described method. BI-MAR and FSMAR reduced the error almost as well to 76 HU respectively 67 HU. Noise was reduced by the described method the most, from 197 HU to 34 HU, but this, again, was due to the averaging (or rather, median) over several scans. LI-MAR and FSMAR perform equally well at 38 HU respectively 37 HU. BI-MAR and FSMAR yielded noise levels of 63 HU amd 48 HU respectively. The visually more compelling reduction of artifacts and noise of the described method was not fully reflected in these figures.

### Computing times and memory demand

**Figure 11:**
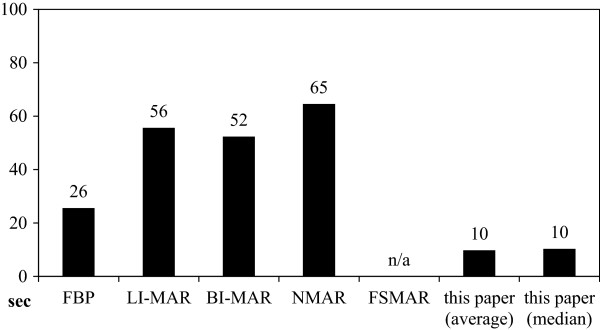
**Computing times.** Computing times in case of established MAR algorithms are dominated by forward- and back-projections. Frequency split MAR (FSMAR) in addition requires a Gaussian blur filter which was excluded from the comparison due to an inefficient implementation for fairness. The MAR method described in this paper did not require any forward- or back-projections and features a low overall computing time.

In case of the described method, computing time was dominated by the co-registration (one matrix-vector coordinate-transformation per voxel and available angle) and combination (one average resp. median evaluation per voxel). In case of the compared MAR methods, computing time was dominated by the number of required forward- and backward-projections (few floating point operations per voxel, times the number of projections) of virtual sinograms and metal maps. In case of FS-MAR, computing time was dominated by our inefficient implementation of the Gaussian blur filter, this was excluded from the comparison for fairness. For comparison, forward-projection, filtering and back-projection (FBP) took 26 seconds, see Figure 11. LI-MAR and BI-MAR performed an additional forward-projection of the metal-map and interpolation. LI-MAR required 56 seconds and BI-MAR required 52 seconds. NMAR needed 65 seconds including the normalization operations. The described method required 10 seconds for either the average and the median.

It should be noted that there was some ‘hidden’ computation time in case of the described method, as the reconstruction software of the CT scanner had to calculate each of the reconstructions, before they were even input to the algorithm. Also, the computing time is outweighed in practice by the time it takes to acquire the additional scans.

Memory demand is higher in case of the described method, if all available reconstructions are held in memory simultaneously. The latter is convenient for implementation, but can be avoided.

## Discussion

As proposed, ‘tilted’ CTs taken from different angles of the same object feature complementary information as far as metal artifacts are concerned. Consequently, the combination of such complementary CTs is less prone to artifacts. The method has been tested with two datasets in a clinical setting. In both cases, metal artifacts were reduced, constituting a proof of principle for this approach.

The reduction of bias and noise by up to 95% is significant and substantial. In all circumstances, the median suppressed artifacts more strongly than the mean, and Hounsfield units were more truthfully represented.

An advantage of the method is that it does not require access to proprietary data of the scanning equipment such as sinogram data or the computation of a virtual sinogram. The method works as a purely post-processing step which can be done at any facility independent of the equipment employed or the used methods for reconstruction. This facilitates introduction into the clinical routine a lot as it alleviates a number of safety concerns and does not involve the equipment manufacturer. Also, unlike most algorithms for metal artifact reduction, the method does not rely on any prior about the missing information.

As the method only relies on operations such as median and mean calculations of CTs from the usual and validated workflow, the safety of the method can be easily established and the post-processing step is quite transparent as far as the validity of the calculated Hounsfield units is concerned.

The drawback of the method is the necessity to obtain at least two independent scans from different angles. This could become problematic whenever time is a concern, organ movement is inevitable or patient compliance is limited. Care must be taken that the repositioning of the patient itself does not affect organ placement.

Computing time is less than in case of established MAR algorithms. However, the additional time to perform several scans will be much more of a concern in clinical routine.

The additional scans took minutes rather than seconds to capture, and there was some ‘hidden’ computation cost as the scanner had to reconstruct each of the volumes, before they were even input into the MAR method. It is understood that the described method has limitations due to this overhead effort.The method improves with the number of independent CTs and their relative angle. A substantial improvement by 75%, however, was observed for only three CTs taken at ±22.5° which should be clinically manageable. For geometrical reasons, a CT gantry with a large opening such as the Toshiba Aquilion LB helps in this respect.

Additional radiation exposure need not be a concern. As demonstrated, each of the n scans can be done at 1/n dose. The mean (and to some degree also the median) of the n CTs will feature a similar signal-to-noise ratio as if a single scan at full dose had been performed.

## Conclusions

A simple post-processing method was described which reduced metal artifacts by up to 95%. While the method required one or more complementary scans, it did not rely on any a priori information. As a pure post-processing method it was independent of the actual image acquisition and did not require any particular instrumental setup or access to raw data. The method was computationally inexpensive, but the time necessary to perform additional scans may limit its practicability. Performance of the method was quantitatively comparable to established algorithms and visually superior in a direct comparison. Dose reduction was demonstrated, artifacts could be reduced without compromising total dose to the patient.

## Competing interests

The authors declare that they have no competing interests.

## Authors’ contributions

HB conceived the method, took measurements, analysed the data and drafted the manuscript. MR helped take measurements and helped draft the manuscript. UG and CB helped draft the manuscript. MS assisted with data handling and conversion and helped draft the manuscript. All authors read and approved the final manuscript.
